# Tuberculinum Kochii

**Published:** 1892-09

**Authors:** 


TUBERCULINUM KOCHII.—A laborer in a manufactory, about forty years old, suffered from tuberculosis of both apices of the lungs. Last March he received twelve powders, of which Nos. 1, 5, and 9 contained Tuberculinum, 8th dec., the others Saccharum-lactis. The second, sixth, and tenth day he complained of chills, followed by heat and sweat. The fever was not very high, but always followed the day after the dose of Tuberculinum.—*A. H. Z.*, 21, '91. S. L.

